# Associations between dietary patterns and gene expression profiles of healthy men and women: a cross-sectional study

**DOI:** 10.1186/1475-2891-12-24

**Published:** 2013-02-12

**Authors:** Annie Bouchard-Mercier, Ann-Marie Paradis, Iwona Rudkowska, Simone Lemieux, Patrick Couture, Marie-Claude Vohl

**Affiliations:** 1 Institute of Nutraceuticals and Functional Foods (INAF), Laval University, 2440 Hochelaga Blvd, Quebec G1V 0A6, Canada; 2 Department of Food Science and Nutrition, Laval University, 2425 de l’Agriculture St, Quebec G1K 7P4, Canada; 3 Laboratory of Endocrinology and Genomics, CHUQ, Laval University Hospital Research Center, 2705 Laurier Blvd, Québec, G1V 4G2, Canada

**Keywords:** Dietary patterns, Western dietary pattern, Prudent dietary pattern, Gene expression, Transcriptomics

## Abstract

**Background:**

Diet regulates gene expression profiles by several mechanisms. The objective of this study was to examine gene expression in relation with dietary patterns.

**Methods:**

Two hundred and fifty four participants from the greater Quebec City metropolitan area were recruited. Two hundred and ten participants completed the study protocol. Dietary patterns were derived from a food frequency questionnaire (FFQ) by factor analysis. For 30 participants (in fasting state), RNA was extracted from peripheral blood mononuclear cells (PBMCs) and expression levels of 47,231 mRNA transcripts were assessed using the Illumina Human-6 v3 Expression BeadChips^®^. Microarray data was pre-processed with Flexarray software and analysed with Ingenuity Pathway Analysis (IPA).

**Results:**

Two dietary patterns were identified. The Prudent dietary pattern was characterised by high intakes of vegetables, fruits, whole grain products and low intakes of refined grain products and the Western dietary pattern, by high intakes of refined grain products, desserts, sweets and processed meats. When individuals with high scores for the Prudent dietary pattern where compared to individuals with low scores, 2,083 transcripts were differentially expressed in men, 1,136 transcripts in women and 59 transcripts were overlapping in men and women. For the Western dietary pattern, 1,021 transcripts were differentially expressed in men with high versus low scores, 1,163 transcripts in women and 23 transcripts were overlapping in men and women. IPA reveals that genes differentially expressed for both patterns were present in networks related to the immune and/or inflammatory response, cancer and cardiovascular diseases.

**Conclusion:**

Gene expression profiles were different according to dietary patterns, which probably modulate the risk of chronic diseases.

**Trial Registration:**

NCT: NCT01343342

## Background

With the knowledge acquired by dietary patterns, dietitians can provide their patients or clients dietary recommendations that take into account not only one nutrient but the overall diet. Dietary patterns have been associated with several cardiovascular risk factors such as blood pressure, obesity, serum lipids, and inflammatory markers such as C-reactive protein (CRP) [1-4]. They have also been related to the risk of mortality from cardiovascular diseases and cancer [5]. In a recent systematic review, the Prudent dietary pattern was associated with a reduced risk of stroke and the Western pattern with an increased risk [6]. The Western dietary pattern was associated with an increased risk of colon cancer [7]. Additionally, Meyerhardt et al. [8] have shown an increase in colon cancer recurrence with the Western dietary pattern. Dietary patterns have also been associated with plasma proteomic biomarkers [9]. The Western dietary pattern was positively associated with a group of protein including proteins related to coagulation and lipid metabolism [9]. A few methods to generate dietary patterns, such as factor and cluster analyses, have been described in the literature [10]. The use of dietary patterns assessed by factor analysis has been proven to be a reproducible and valid method among different populations [11-13].

Microarray data can be used to study changes in gene expression for thousands of genes simultaneously. Gene expression studies have observed associations with diseases such as cancer and cardiovascular diseases [14,15]. Diet is an important regulator of gene expression [14,16]. Dietary patterns may impact gene expression through several mechanisms, for example certain dietary compounds bind to transcription factors and regulate their activity such as polyunsaturated fatty acids (PUFA) with *peroxisome proliferator-activated receptors* (*PPARs*) [17]. Studies regarding energy restricted diets and their effects on gene expression levels have observed down-regulation of genes involved in glycolytic and lipid synthesis pathways [18,19]. Distribution of macronutrients also seems to have an impact on gene expression regulation. Compared to a diet rich in monounsaturated fats, a diet rich in saturated fats resulted in a more proinflammatory gene expression profile [20]. The Mediterranean diet has been associated with a decreased in expression of genes involved in the inflammatory response [21].

To our knowledge, the effects of dietary patterns derived from factor analysis on gene expression profile have never been investigated. Thus, the objective of this study was to examine associations between dietary patterns derived from factor analysis and gene expression profiles.

## Methods

### Subjects and study design

Two hundred and fifty four participants were recruited between September 2009 and December 2011 from the greater Quebec City metropolitan area through advertisements in local news as well as by electronic messages sent to university students/employees. Women who were pregnant or breastfeeding were excluded. To be eligible, participants had to be between 18 to 50 years of age, non-smokers and free of any thyroid or metabolic disorders requiring treatment, such as diabetes, hypertension, severe dyslipidemia and coronary heart disease requiring treatment. The body mass index (BMI) of the participants was between 25 and 40 kg/m^2^. Subjects drinking regularly more than 2 drinks per day, taking omega-3 PUFA (n-3 PUFA) supplements 6 months prior to the study and other medication or supplement affecting lipid and lipoprotein metabolism were excluded. A total of 210 participants completed the protocol which is described elsewhere [22] and were included in this cross-sectional study. Subjects all provided written consent to participate into the study, which was approved by the ethics committees of Laval University Hospital Research Center and Laval University. This trial was registered at clinicaltrials.gov as NCT01343342.

### Anthropometric measurements

Body weight, height, and waist circumference were measured according to the procedures recommended by the Airlie Conference [23]. BMI was calculated as weight per meter squared (kg/m^2^).

### Biochemical parameters

The morning after a 12-hour overnight fast and 48-h alcohol abstinence, blood samples were collected from an antecubital vein into vacutainer tubes containing EDTA. Blood samples were used to identify individuals with metabolic disorders, which were excluded. Plasma was separated by centrifugation (2500 × g for 10 minutes at 4°C), samples were aliquoted and frozen for subsequent analyses. Plasma total cholesterol (TC) and triglyceride concentrations were measured using enzymatic assays [24,25]. Infranatant (d >1.006 g/ml) with heparin-manganese chloride was used to precipitate very low-density lipoprotein (VLDL) and low-density lipoprotein (LDL) and then determine high-density lipoprotein (HDL) cholesterol concentrations (HDL-C) [26]. The equation of Friedewald was used to estimate LDL-cholesterol (LDL-C) [27]. Non-HDL-C was calculated by subtracting HDL-C from TC. CRP was measured by nephelometry (Prospec equipment Behring) using a sensitive assay, as described previously [28]. Plasma apolipoprotein B-100 (apoB) concentrations were measured by the rocket immunoelectrophoretic method of Laurell, as previously described [29]. Glucose concentrations were determined enzymatically [30] and plasma insulin was measured by radioimmunoassay with polyethylene glycol separation [31].

### Blood pressure measurements

Resting blood pressure measurements (three readings) were performed after a 10-min rest in a sitting position, phases I and V of Korotkoff sounds being respectively used for systolic (SBP) and diastolic (DBP) blood pressures [32].

### Dietary assessment and food grouping

Dietary intake of the past month was determined by a 91-items validated food frequency questionnaire (FFQ) [33] based on food habits of Quebecers, administered by a registered dietitian (RD). The RD asked participants how often they consumed each type of food: daily, weekly, monthly or none at all during the last month. To make sure each participant estimated correctly the portion eaten, examples of portion size were provided. Data obtained from FFQ were analysed using the Nutrition Data System for Research software version 2011, developed by the Nutrition Coordination Center (University of Minnesota, Minneapolis, MN). All the information was compiled and similar food items from the FFQ were grouped, as previously described [4]. Three criteria were used to form these groups: first, the similarity of nutrient profiles, second, the culinary usage of different types of food (similar to groups used in a previous study [11]) and third, the consideration of groups utilized in other studies to maintain consistency [34]. Some individual food items were classified separately when their composition differed considerably from other foods (for example, pizza or eggs) or when they represented a different dietary habit (for example, liquor, wine, beer and French fries). On this basis, thirty-seven food groups were formed as described by Paradis et al. [4]. Food items from only thirty-five food groups were consumed by the participants in the present study. From these thirty-five food groups, eight were not normally distributed even after logarithmic transformation and were excluded as well. Consequently, twenty-seven foods groups were used for factor analyses to generate dietary patterns.

### Food pattern derivation

Food patterns derivation methods have already been described in a previous study [4]. Briefly, variables with abnormal distribution where logarithmically transformed before further analyses. The FACTOR procedure from Statistical Analysis Software (SAS) was used to derive factors. To determine the number of factors to retain, components with eigenvalue > 1, values at Scree test and the interpretability were considered. Food groups with absolute factor loadings ≥ 0.30 were regarded as significant contributors to the pattern. The patterns (derived factors) were named according to the interpretation of the data and to previous literature [4]. Each participant was given a score for both dietary patterns. These scores were calculated from the sum of food groups multiplied by their respective factor loading with the SCORE procedure of SAS. These scores reflect the degree of each participant dietary intakes conforming to a dietary pattern. In order to form two groups for each dietary pattern, participants were divided according to their score. A score ≤ 0 was considered as «low» and a score > 0 was considered as «high».

### Transcriptomics analyses

For transcriptomics analyses, the first 30 individuals who completed the study were included (13 men and 17 pre-menopausal women). In human nutrition studies 5 to 10 individuals in each group appear to be sufficient to detect differently expressed genes [35]. The following methods have been described by Rudkowska et al. [22]. Briefly, peripheral blood mononuclear cells (PBMCs) were collected into an 8-ml Cell Preparation Tube (Becton Dickinson, Oakville, Ontario, Canada). Remarkable concordance (<80%) of gene expression profiles between PBMCs and different tissues including liver, kidney, stomach, spleen, prostate, lung, heart, colon and brain, has been reported [36]. Centrifugation at room temperature (1500g, 20 minutes) was executed to separate PBMCs. The RNeasy Plus Mini Kit (QIAGEN, Mississauga, Ontario, Canada) was used to extract total RNA according to the manufacturer’s protocol. Microarray analyses were performed after spectrophotometric quantification and verification of the total RNA quality on the Agilent 2100 Bioanalyser (Agilent Technologies, Palo Alto, CA, United States). None of the samples had RNA integrity number (RIN) values less than 8 [37]. Consequently, all samples were included in the microarray analysis. However, as described previously [22], one outlier was excluded due to abnormal hybridization results. Thus, further analyses were conducted with 29 participants. The Illumina TotalPrep RNA Amplification kit (Ambion, Austin, TX, United States) was used to amplify and label 200ng of total RNA. The quality of complimentary RNA (cRNA) was evaluated by capillary electrophoresis on Agilent 2100 Bioanalyzer. 37,804 genes were analysed via expression levels of 48,803 mRNA transcripts with the Human-6 v3 Expres-sion BeadChips^®^ (Illumina, San Diego, CA, United States). The McGill University and Génome Québec Innovation Center (Montreal, Quebec, Canada) performed hybridization according to the manufacturer’s instructions, as previously described [22]. Validation of the expression levels were assessed previously by polymerase chain reaction (PCR) [22].

### Analysis of microarray data

Microarray data was analysed with Flexarray software [38]. The lumi Bioconductor package algorithm included in Flexarray software, was used to pre-process and normalize Illumina microarray data. Background correction was assessed using negative controls followed by log_2_ to stabilize variance and quantile normalization. According to Shi et al. [39] the use of control probes during background correction minimize false discovery rate (FDR). Fold changes obtained with this method also relate more to fold changes observed with PCR validation tests [39]. Participants where then stratified according to sex and scores for Prudent and Western dietary patterns. To assess differences (separately for men and for women) in gene expression levels between high versus low scores for Prudent and Western dietary patterns, the Significant Analysis of Microarray (SAM) was performed. SAM is an adaptation of *t*-test for microarray data which assigns a score to each gene according to changes in gene expression relative to the standard deviation of repeated measurements [40]. SAM uses permutation of the repeated measurements to estimate FDR. Transcripts were declared differently expressed only when P < 0.05 and fold changes were either < 0.8 (down-regulated) or > 1.2 (up-regulated), as previously described [22].

### Biological pathway analyses

Ingenuity Pathway Analysis (IPA) system (Ingenuity^®^ System, http://www.ingenuity.com) was used to verify if differentially expressed genes were related more than expected by chance to networks, diseases and canonical pathways. IPA allows adding structure to the vast amount of data generated by microarrays. To begin, an input file containing fold changes and P values of all probe sets was uploaded into IPA system. From this file, dataset in Core Analysis was produced. General settings for IPA system as «Ingenuity^®^ Knowledge Base (genes)» and «considered only molecules and/or relationships for humans» were used. IPA calculates a P value based on the right-tailed Fisher’s exact test for each canonical pathway, which is a measure of the likelihood that the association of a data set with a pathway is due to random chance. A cutoff of 1.2 was set. Relevant pathways with P values smaller than 0.05, were taken into account. IPA suggests that canonical pathways with P values higher than 0.05 may also be biologically relevant. Furthermore, no direction is associated with pathways, in other words, they cannot be qualified as up- or down-regulated. Significantly relevant canonical pathways related to cancer, cardiovascular diseases, immune system and inflammation were considered. IPA also processes «Downstream Effects Analysis» which is based on expected causal effects between genes and functions. These causal effects are derived from literature and compiled in «Ingenuity^®^ Knowledge Base». «Downstream Effects Analysis» compares the direction of the differently expressed genes with expectations based on the literature and predicts for each function a direction change using the «regulation z-score algorithm». In other words, if the observed direction change is mostly consistent with a particular activation state (increase or decrease) then IPA make a directional prediction. The z-score algorithm is designed to reduce the chance that random data will generate significant predictions. Z-scores ≥ 2, indicate that the function is significantly increased and z-scores ≤ -2, indicate that the function is significantly decreased. IPA also calculates a P value with the Fisher’s Exact Test which represents the likelihood that the association between a set of genes from the experimental data set and a related function is due to random association.

### Statistical analyses

Comparisons between individuals with high and low dietary pattern scores were performed through the General Linear Model (GLM) procedure and using the type 3 sum of squares (for unbalanced study design). Variables with abnormal distribution were logarithmically transformed. Age, BMI and energy intakes (for all dietary intakes, except energy intakes) were included as potential confounders. Statistical significance was defined as *P* < 0.05. Statistical analyses were performed with SAS statistical package (version 9.2; SAS Institute, Inc., Cary, NC, USA).

## Results

Two main dietary patterns were derived from factor analysis. Factor loadings for both dietary patterns are listed in Table 1. A factor loading ≥ 0.30 indicates a strong positive association with the dietary pattern whereas a factor loading ≤ -0.30 indicates a strong inverse association with the dietary pattern. The Prudent dietary pattern was positively associated with vegetables, fruits, whole grain products food groups and inversely associated with refined grain products food group. The Western dietary pattern was positively associated with refined grain products, desserts, sweets and processed meat food groups.

**Table 1:** Factor loadings for Prudent and Western dietary patterns (n = 210)

**Food groups (servings/day)**	Factor 1* Prudent	Factor 2* Western
Vegetables	**0.71**	0.03
Fruits	**0.60**	-0.01
Whole grain products	**0.53**	0.21
Non-hydrogenated fat	**0.46**	0.02
Refined grain products	**-0.45**	**0.39**
Desserts	-0.01	**0.80**
Sweets	0.09	**0.77**
Beer	0.01	-0.03
Coffee	0.06	0.15
Poultry	-0.004	-0.06
Red meat	-0.11	0.11
Potatoes other than French fries	0.09	0.16
Processed meat	-0.10	**0.33**
Legumes	0.15	0.13
Tea	0.08	-0.02
High-fat dairy products	0.13	0.13
Low-fat dairy products	0.27	0.07
Eggs	0.27	-0.05
Cream soup	-0.11	0.12
Pizza	-0.23	-0.03
Fish and other sea food	0.28	-0.03
Fruit juices	-0.14	0.02
Nuts	0.26	0.06
Vegetable juices	0.12	0.05
Condiments	0.18	0.06
Snacks	-0.11	0.18
Saturated fat (butter and lard)	0.04	0.06
Variance explained (%)	12.96	10.62

*Exploratory factor analysis using the FACTOR procedure.

Factor loading ≥ 0.30 or ≤ -0.30 are marked in bold.

Descriptive characteristics of the 29 study participants are presented separately for men and women in Table 2 and Table 3 according to low (≤0) or high (>0) scores for both dietary patterns. Fasting insulin concentrations were significantly (P = 0.03) lower in men with high scores for the Prudent dietary pattern, as compared to men with low scores for the Prudent dietary pattern. Also, men with a high score for the Western dietary pattern had a significantly higher systolic and diastolic blood pressure (P = 0.0008 and P = 0.01, respectively) than men with a low scores and a trend (P = 0.05) was observed for higher fasting glucose concentrations. Women with a high score for the Western dietary pattern had higher fasting glucose concentrations (P = 0.03) than those with a low score. When comparing cardiovascular risk factors for women with high scores versus low scores for the Prudent dietary pattern, only trends were observed. Women with high scores for the Prudent dietary pattern had a higher BMI (P = 0.09) but lower plasma triglyceride concentrations (P = 0.07). When comparing mean dietary intakes of men (n = 12) between women (n = 17), men had significantly higher total fat (33.85% ± 4.16% and 29.81% ± 3.62% respectively, P = 0.01) and monounsaturated fat intakes (14.40% ± 1.94% versus 12.11% ± 1.79% respectively, P = 0.004) than women, independently of age and BMI. Saturated fat, polyunsaturated fat and total fiber intakes were not significantly different when age and BMI were included as potential confounders. For the entire cohort (210 participants), only associations for the Prudent pattern were observed. Men (n = 97) with high scores had lower fasting insulin (P = 0.04) and glucose concentrations (P = 0.003) as compared to men with low scores (data not shown). Women (n = 113) with high Prudent dietary pattern scores had lower apoB (P = 0.04) and TC (P = 0.04) concentrations (data not shown).

**Table 2:** Descriptive characteristics of the study participants for men according to dietary pattern scores

	**Prudent dietary pattern (n = 12)**		***P***	**Western dietary pattern (n = 12)**		***P***
	**Low score (n = 5)**	**High score (n = 7)**		**Low score (n = 3)**	**High score (n = 9)**	
**Age (y)**	34.40 ± 10.99	32.71 ± 5.88	0.74	31.00 ± 2.65	34.22 ± 9.09	0.57
**BMI (kg/m**^**2**^**)**	28.86 ± 3.62	29.63 ± 5.56	0.88^1^	32.04 ± 8.04	28.40 ± 3.18	0.35^1^
**Waist circumference (cm)**	96.83 ± 9.24	95.53 ± 12.86	0.45^2^	102.87 ± 15.26	93.81 ± 9.27	0.68^2^
**Systolic blood pressure (mmHg)**	113.80 ± 5.89	107.86 ± 7.24	0.18^2^	101.67 ± 2.08	113.22 ± 5.56	0.0008^2^
**Diastolic blood pressure (mmHg)**	76.40 ± 2.70	68.86 ± 8.32	0.12^2^	63.33 ± 1.53	74.89 ± 6.17	0.01^2^
**Fasting glucose (mmol/L)**	5.20 ± 0.31	4.83 ± 0.63	0.32^2^	4.40 ± 0.46	5.18 ± 0.41	0.05^2^
**Fasting insulin (pmol/L)**	123.00 ± 63.49	73.29 ± 23.56	0.03^2^	73.67 ± 16.65	100.78 ± 55.29	0.14^2^
**CRP (mg/L)**	4.33 ± 5.81	2.02 ± 2.24	0.47^2^	2.88 ± 2.92	3.02 ± 4.52	0.92^2^
**Total-C (mmol/L)**	5.77 ± 0.85	5.08 ±1.04	0.33^2^	5.60 ± 0.98	5.29 ± 1.04	0.57^2^
**LDL-C (mmol/L)**	3.69 ± 0.55 (n = 4)	3.18 ± 0.97	0.47^2^	3.74 ± 0.60	3.22 ± 0.92 (n = 8)	0.33^2^
**HDL-C (mmol/L)**	1.09 ± 0.22	1.33 ± 0.39	0.23^2^	1.10 ± 0.35	1.27 ± 0.35	0.62^2^
**Triglycerides (mmol/L)**	2.48 ± 1.88	1.24 ± 0.54	0.20^2^	1.65 ± 0.29	1.79 ± 1.59	0.92^2^
**ApoB (g/L)**	1.14 ± 0.23	0.94 ± 0.27	0.26^2^	1.14 ± 0.11	0.98 ± 0.29	0.38^2^
**Dietary intakes**						
**Total fat (%)**	34.22 ± 2.23	33.58 ± 5.32	0.78^2^	32.52 ± 7.43	34.29 ± 3.02	0.40^2^
**Saturated fat (%)**	12.24 ± 1.47	9.77 ± 2.43	0.09^2^	8.57 ± 3.34	11.54 ± 1.57	0.03^2^
**Monounsaturated fat (%)**	14.34 ± 0.85	14.44 ± 2.53	0.97^2^	14.44 ± 3.06	14.38 ± 1.68	0.93^2^
**Polyunsaturated fat (%)**	4.98 ± 0.57	6.61 ± 1.50	0.08^2^	6.57 ± 0.91	5.72 ± 1.56	0.49^2^
**Total fiber (g)**	19.40 ± 8.31	31.50 ± 7.53	0.03^2^	26.04 ± 2.29	26.60 ± 11.36	0.82^2^

All values are means ± SDs. Tests for trends or differences were made by using generalized linear models.

^1^ Adjusted for age.

^2^ Adjusted for age and BMI.

**Table 3:** Descriptive characteristics of the study participants for women according to dietary pattern scores

	**Prudent dietary pattern (n = 17)**		***P***	**Western dietary pattern (n = 17)**		***P***
	**Low score (n = 10)**	**High score (n = 7)**		**Low score (n = 11)**	**High score (n = 6)**	
**Age (y)**	34.80 ± 11.16	33.86 ± 9.51	0.86	35.45 ± 9.78	32.50 ± 11.64	0.59
**BMI (kg/m**^**2**^**)**	28.09 ± 2.71	30.94 ± 3.58	0.09^1^	29.01 ± 2.73	29.73 ± 4.48	0.68^1^
**Waist circumference (cm)**	82.82 ± 8.07	91.14 ± 7.87	0.31^2^	86.05 ± 8.85	86.60 ± 9.58	0.88^2^
**Systolic blood pressure (mmHg)**	106.40 ± 8.42	103.57 ± 8.92	0.14^2^	103.45 ± 9.29	108.50 ± 6.12	0.11^2^
**Diastolic blood pressure (mmHg)**	67.70 ± 8.84	72.00 ± 8.79	0.91^2^	68.73 ± 9.23	70.83 ± 8.66	0.57^2^
**Fasting glucose (mmol/L)**	5.20 ± 1.08	4.66 ± 0.58	0.30^2^	4.74 ± 0.42	5.42 ± 1.42	0.03^2^
**Fasting insulin (pmol/L)**	83.10 ± 23.32	77.71 ± 31.39	0.15^2^	78.82 ± 27.99	84.67 ± 24.31	0.78^2^
**CRP (mg/L)**	7.03 ± 12.01	6.14 ± 9.90	0.51^2^	6.65 ± 11.32	6.69 ± 11.01	0.41^2^
**Total-C (mmol/L)**	5.37 ± 1.55	4.95 ± 1.23	0.85^2^	5.04 ± 1.35	5.48 ± 1.58	0.35^2^
**LDL-C (mmol/L)**	3.06 ± 1.12	2.76 ± 1.20	0.92^2^	2.90 ± 1.17	3.01 ± 1.15	0.59^2^
**HDL-C (mmol/L)**	1.64 ± 0.66	1.68 ± 0.39	0.49^2^	1.52 ± 0.47	1.91 ± 0.64	0.14^2^
**Triglycerides (mmol/L)**	1.48 ± 0.77	1.09 ± 0.52	0.07^2^	1.36 ± 0.72	1.25 ± 0.69	0.64^2^
**ApoB (g/L)**	0.98 ± 0.28	0.81 ± 0.29	0.37^2^	0.90 ± 0.28	0.94 ± 0.33	0.63^2^
**Dietary intakes**						
**Total fat (%)**	29.17 ± 4.02	30.73 ± 3.00	0.61^2^	28.55 ± 3.59	32.13 ± 2.50	0.08^2^
**Saturated fat (%)**	10.45 ± 1.54	9.45 ± 2.32	0.22^2^	9.32 ± 1.96	11.35 ± 0.81	0.05^2^
**Monounsaturated fat (%)**	11.45 ± 1.84	13.04 ± 1.33	0.13^2^	11.67 ± 1.91	12.90 ± 1.36	0.28^2^
**Polyunsaturated fat (%)**	4.81 ± 0.81	5.71 ± 0.69	0.05^2^	5.14 ± 0.98	5.26 ± 0.70	0.90^2^
**Total fiber (g)**	19.78 ± 3.88	28.14 ± 6.77	0.03^2^	24.14 ± 7.11	21.54 ± 5.77	0.36^2^

All values are means ± SDs. Tests for trends or differences were made by using generalized linear models.

^1^ Adjusted for age.

^2^ Adjusted for age and BMI.

Intakes of the most associated food groups with the dietary patterns are presented in Table 4 and Table 5. Men with high scores for the Prudent dietary pattern had higher intakes of vegetables (P = 0.03), fruits (P = 0.02), whole grain products (P = 0.004), fish (P = 0.04), and nuts (P = 0.02) than men with low scores. Women with high scores for the Prudent dietary pattern had higher intakes of non-hydrogenated fats (P = 0.006), and lower intakes of sweets (P = 0.05) than women with low scores. For the Western dietary pattern, both men and women with high scores had higher intakes of sweets than individuals with low scores (P = 0.03 and P = 0.007, respectively). Only for the women, the intake of desserts (P < 0.0001) was significantly higher with a high Western dietary pattern score. The men with high scores for the Western dietary pattern had lower intakes of vegetables (P = 0.03).

**Table 4:** Food group intakes (number of servings) for men according to dietary pattern scores

	**Prudent dietary pattern (n = 12)**		***P***	**Western dietary pattern (n = 12)**		***P***
	**Low score (n = 5)**	**High score (n = 7)**		**Low score (n = 3)**	**High score (n = 9)**	
Vegetables*	1.90 ± 0.65	3.93 ± 1.80	0.03^1^	4.47 ± 2.57	2.62 ± 1.23	0.03^1^
Fruits*	1.34 ± 1.18	3.20 ± 0.95	0.02^1^	2.98 ± 0.50	2.24 ± 1.55	0.28^1^
Whole grain products*	1.82 ± 1.43	4.37 ± 1.35	0.004^1^	3.33 ± 0.96	3.30 ± 2.12	0.35^1^
Non-hydrogenated fats*	4.02 ± 4.19	4.58 ± 3.02	0.75^1^	3.96 ± 2.58	4.47 ± 3.74	0.28^1^
Refined grain products*	3.26 ± 2.33	2.27 ± 1.30	0.23^1^	1.40 ± 1.25	3.10 ± 1.77	0.38^1^
Fish and other sea food*	0.61 ± 0.66	1.71 ± 0.88	0.04^1^	1.22 ± 0.87	1.26 ± 1.03	0.60^1^
Nuts*	0.33 ± 0.13	1.88 ± 1.88	0.02^1^	1.24 ± 0.68	1.23 ± 1.85	0.38^1^
Desserts*	1.03 ± 0.43	0.86 ± 0.85	0.75^1^	0.36 ± 0.52	1.12 ± 0.65	0.21^1^
Sweets*	4.86 ± 2.07	4.35 ± 2.60	0.60^1^	1.86 ± 1.17	5.47 ± 1.82	0.03^1^
Processed meats*	0.96 ± 0.91	0.72 ± 0.76	0.59^1^	0.12 ± 0.20	1.06 ± 0.78	0.16^1^

All values are means ± SDs. Tests for trends or differences were made by using generalized linear models.

^1^Adjusted for age, BMI and energy intakes.

*Vegetables, 1 serving = 125 ml of all vegetables; Fruits, 1 serving = 1 unit of fresh fruit, 125 ml of fruit compote (stewed), frozen and canned or 60 ml of dried fruit; Whole grain products, 1 serving = 1 unit of whole-wheat, whole-grain or other multigrain breads, ½ unit of whole-wheat, whole-grain and other multigrain bagels, tortillas and pitas, 125 ml of whole-wheat or whole-grain pasta, 125 ml of brown rice, 125 ml of oatmeal and wheat cream, 30 g of whole-grain cereal, 1 unit of whole wheat pancakes, 30 g of whole-wheat crackers; Non-hydrogenated fats, 1 serving = 5 ml of non-hydrogenated margarine, 5 ml of vegetable oil or 5 ml of salad dressing (all kinds); Refined grain products, 1 serving = 1 unit of white breads, ½ unit of white bagels, pitas and tortillas, 125 ml of white rice, white pasta and couscous, 1 unit of muffins (home-made), pancakes, waffles and granola bars, 30 g of refined cereal, 1 unit of rice cake or 30 g of salted crackers; Fish and other sea food, 1 serving = 30 g of fish and sea food (excluding breaded fish) or 1 unit of sushi; Nuts, 1 serving = 30 ml of all nuts and seeds or 30 ml of seed and nuts butter; Desserts, 1 serving = 2 units of cookies, 1/6 pies, 125 ml of pudding, 1/6 or 1 unit of cakes, doughnuts and pastries, 1 unit of croissants, muffins (commercial), date squares, banana breads and coated granola bars; Sweets, 1 serving = 5 ml of sugar, brown sugar, honey, maple taffy, jam, maple/corn syrup and nutella, 1 unit of candy, 5 g of chocolate bar or pieces; Processed meats, 1 serving = 5 slices of bacon, 1 unit of sausage, hotdogs and hotdogs on a stick, 30 g delicatessen, cretons, head cheese, liver pâté and terrine.

**Table 5:** Food group intakes (number of servings) for women according to dietary pattern scores

	**Prudent dietary pattern (n = 12)**		***P***	**Western dietary pattern (n = 12)**		***P***
	**Low score (n = 10)**	**High score (n = 7)**		**Low score (n = 3)**	**High score (n = 9)**	
Vegetables*	2.30 ± 1.55	4.38 ± 1.73	0.16^1^	3.09 ± 2.05	3.28 ± 1.73	0.77^1^
Fruits*	1.97 ± 1.14	3.34 ± 0.61	0.07^1^	2.56 ± 1.21	2.48 ± 1.18	0.62^1^
Whole grain products*	2.04 ± 1.33	4.02 ± 2.84	0.18^1^	3.25 ± 2.47	2.13 ± 1.70	0.13^1^
Non-hydrogenated fats*	1.52 ± 0.97	4.65 ± 3.09	0.006^1^	2.58 ± 2.66	3.24 ± 2.59	0.96^1^
Refined grain products*	3.14 ± 1.50	1.83 ± 0.61	0.06^1^	2.46 ± 1.31	2.86 ± 1.53	0.57^1^
Fish and other sea food*	1.01 ± 0.72	1.24 ± 0.88	0.35^1^	0.85 ±0.52	1.58 ± 0.96	0.11^1^
Nuts*	0.39 ± 0.39	0.59 ± 0.63	0.89^1^	0.56 ± 0.57	0.30 ± 0.30	0.29^1^
Desserts*	0.85 ± 0.75	0.62 ± 0.38	0.38^1^	0.38 ± 0.28	1.44 ± 0.42	<0.0001^1^
Sweets*	3.45 ± 1.69	2.93 ± 5.05	0.05^1^	1.90 ± 1.60	5.69 ± 4.43	0.007^1^
Processed meats*	0.38 ± 0.37	0.30 ± 0.17	0.13^1^	0.31 ± 0.28	0.43 ± 0.36	0.67^1^

All values are means ± SDs. Tests for trends or differences were made by using generalized linear models.

^1^Adjusted for age, BMI and energy intakes.

*Vegetables, 1 serving = 125 ml of all vegetables; Fruits, 1 serving = 1 unit of fresh fruit, 125 ml of fruit compote (stewed), frozen and canned or 60 ml of dried fruit; Whole grain products, 1 serving = 1 unit of whole-wheat, whole-grain or other multigrain breads, ½ unit of whole-wheat, whole-grain and other multigrain bagels, tortillas and pitas, 125 ml of whole-wheat or whole-grain pasta, 125 ml of brown rice, 125 ml of oatmeal and wheat cream, 30 g of whole-grain cereal, 1 unit of whole wheat pancakes, 30 g of whole-wheat crackers; Non-hydrogenated fats, 1 serving = 5 ml of non-hydrogenated margarine, 5 ml of vegetable oil or 5 ml of salad dressing (all kinds); Refined grain products, 1 serving = 1 unit of white breads, ½ unit of white bagels, pitas and tortillas, 125 ml of white rice, white pasta and couscous, 1 unit of muffins (home-made), pancakes, waffles and granola bars, 30 g of refined cereal, 1 unit of rice cake or 30 g of salted crackers; Fish and other sea food, 1 serving = 30 g of fish and sea food (excluding breaded fish) or 1 unit of sushi; Nuts, 1 serving = 30 ml of all nuts and seeds or 30 ml of seed and nuts butter; Desserts, 1 serving = 2 units of cookies, 1/6 pies, 125 ml of pudding, 1/6 or 1 unit of cakes, doughnuts and pastries, 1 unit of croissants, muffins (commercial), date squares, banana breads and coated granola bars; Sweets, 1 serving = 5 ml of sugar, brown sugar, honey, maple taffy, jam, maple/corn syrup and nutella, 1 unit of candy, 5 g of chocolate bar or pieces; Processed meats, 1 serving = 5 slices of bacon, 1 unit of sausage, hotdogs and hotdogs on a stick, 30 g delicatessen, cretons, head cheese, liver pâté and terrine.

**Figure 1:**
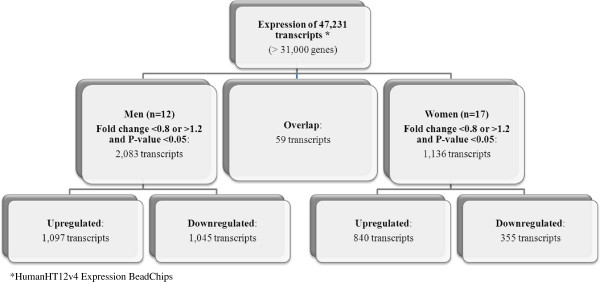
Flowchart illustrating the significantly different transcripts according to scores for the Prudent dietary pattern.

**Figure 2:**
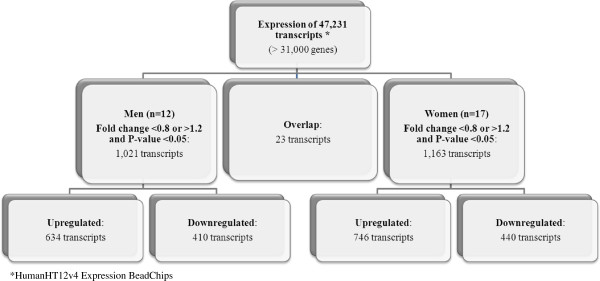
Flowchart illustrating the significantly different transcripts according to scores for the Western dietary pattern.

As previously described [22], approximately 55% of transcripts were detected in the PBMCs of study participants. As shown in Figure 1, when individuals with high scores for the Prudent dietary pattern where compared to individuals with low scores, 2,083 transcripts were differentially expressed in men, 1,136 transcripts in women and 59 transcripts were overlapping. In men comparing high scores to low scores for the Prudent dietary pattern, 1,045 transcripts were down-regulated (49%) and 1,097 were up-regulated (51%). For the women, 355 transcripts were down-regulated (30%) and 840 were up-regulated (70%). As shown in Figure 2, for the Western dietary pattern, 1,021 transcripts were differentially expressed in men with high versus low scores, 1,163 transcripts were differentially expressed in women and 23 transcripts were overlapping in men and women. In men comparing high scores to low scores for the Western dietary pattern, 410 transcripts were down-regulated (39%) and 634 were up-regulated (61%). For women comparing high scores to low scores for the Western dietary pattern, 440 transcripts were down-regulated (37%) and 746 were up-regulated (63%).

**Figure 3:**
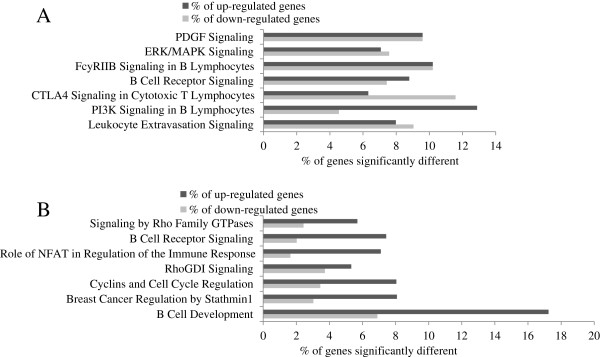
**The modified canonical pathways according to scores for the Prudent dietary pattern. Legend:** Gene expression differences (≥ ± 1.2 fold change) in canonical pathways comparing high and low scores for the Prudent dietary pattern **(A)** For the men **(B)** For the women. P-values for Functional Analysis of dataset by IPA (Fisher’s Exact Test) are presented. % of genes significantly up-regulated and down-regulated in each canonical pathway are presented (number of genes differently expressed/number of genes in the pathways*100). **(A)** PDGF Signaling: P = 0.004, 14 genes significantly different; ERK/MAPK Signaling: P = 0.004, 29 genes significantly different; FcyRIIB Signaling in B Lymphocytes: P = 0.003, 10 genes significantly different; B Cell Receptor Signaling: P = 0.002, 24 genes significantly different; CTLA4 Signaling in Cytotoxic T Lymphocytes: P = 0.003, 17 genes significantly different; PI3K Signaling in B Lymphocytes: P = 0.0009, 23 genes significantly different; Leucocyte Extravasation Signaling: P = 0.0002, 32 genes significantly different. **(B)** Signaling by Rho Family GTPase: P = 0.02, 20 genes significantly different; B Cell Receptor Signaling: P = 0.02, 14 genes significantly different; Role of NFAT in Regulation of the Immune Response: P = 0.01, 16 genes significantly different; RhoGDI Signaling: P = 0.009, 17 genes significantly different; Cyclins and Cell Cycle Regulation: P = 0.007, 10 genes significantly different; Breast Cancer Regulation by Stathmin1: P = 0.0005, 22 genes significantly different; B Cell Development: P = 0.0004, 7 genes significantly different.

**Figure 4:**
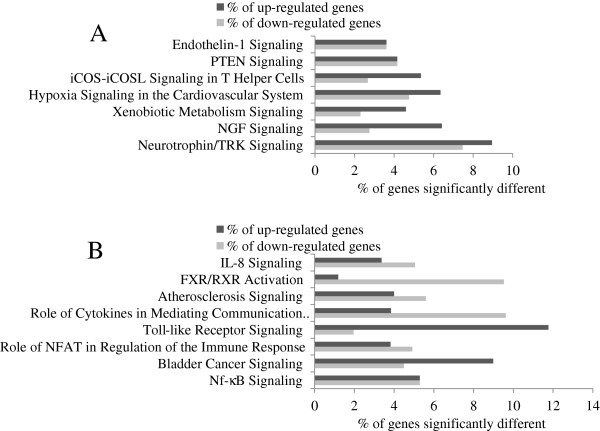
**The modified canonical pathways according to scores for the Western dietary pattern. Legend:** Gene expression differences (≥ ± 1.2 fold change) in canonical pathways comparing high and low scores for the Western dietary pattern **(A)** For the men **(B)** For the women. P-values for Functional Analysis of dataset by IPA (Fisher’s Exact Test) are presented. % of genes significantly up-regulated and down-regulated in each canonical pathway are presented (number of genes differently expressed/number of genes in the pathways*100). **(A)** Endothelin-1 Signaling: P = 0.01, 12 genes significantly different; PTEN Signaling: P = 0.01, 10 genes significantly different; iCOS-iCOSL Signaling in T Helper Cells: P = 0.008, 9 genes significantly different; Hypoxia Signaling in the Cardiovascular System: P = 0.006, 7 genes significantly different; Xenobiotic Metabolism Signaling: P = 0.005, 18 genes significantly different; NGF Signaling: P = 0.004, 10 genes significantly different; Neurotrophin/TRK Signaling: P = 0.00001, 11 genes significantly different. **(B)** IL-8 Signaling: P = 0.04, 15 genes significantly different; FXR/RXR Activation: P = 0.05, 9 genes significantly different; Atherosclerosis Signaling: P = 0.02, 12 genes significantly different; Role of Cytokines in Mediating Communication between Immune Cells: P = 0.02, 7 genes significantly different; Toll-like Receptor Signaling: P = 0.01, 7 genes significantly different; Role of NFAT in Regulation of the Immune Response: P = 0.01, 16 genes significantly different; Bladder Cancer Signaling: P = 0.002, 12 genes significantly different; Nf-κB Signaling: P = 0.002, 18 genes significantly different.

According to IPA, few canonical pathways were significantly modified in men and women when comparing high scores to low scores for both dietary patterns (Figure 3(A), (B), Figure 4(A) and (B)).

Interestingly, IPA was able to predict an activation state for different functions when comparing high scores to low scores for the Prudent dietary pattern. In men, a decrease in colony formation (z-score -2.10; 40 molecules; P = 0.009), decreased adhesion of prostate cancer cell lines (z-score -2.17; 6 molecules; P = 0.01), increased cell death of connective tissue cells (z-score 2.69; 19 molecules; P = 0.01) and smooth muscle cells (z-score 2.55; 6 molecules; P = 0.03), an increase in the metabolism of phosphatidic acid (PA) (z-score 2.23; 14 molecules; P = 0.04), of phospholipid (z-score 2.22; 17 molecules; P = 0.04) and in apoptosis of connective tissue cells (z-score 2.77; 13 molecules; P = 0.04) were predicted. For women, IPA predicted a decreased tumorigenesis (z-score -2.30; 258 molecules; P = 0.0001), mitosis of tumor cell lines (z-score -2.39; 11 molecules; P = 0.003), tyrosine phosphorylation (z-score -2.03; 13 molecules; P = 0.003) and survival of tumor cell lines (z-score -2.11; 33 molecules; P = 0.03). When comparing high scores to low scores for the Western dietary pattern in men, IPA predicted a decrease in apoptosis of tumor cell lines (z-score -3.42; 54 molecules; P = 0.005), a decrease in cell death of tumor cell lines (z-score -2.73; 57 molecules; P = 0.08), an increase in quantity of PA (z-score 2.11; 5 molecules; P = 0.02) and of carbohydrate (z-score 2.31; 6 molecules; P = 0.04). For women according to the Western dietary pattern, IPA was unable to make a prediction for the activation state of any function.

## Discussion

The dietary patterns derived in this study resemble «Prudent» and «Western» dietary patterns from the literature [41]. The Prudent dietary pattern is usually associated with vegetables, fruits, whole grain products, fish and non-hydrogenated fats [4,42], whereas the Western dietary pattern is described as high in red meats, processed meats, refined grains, French fries and sweets/desserts [4,42]. Participants with high scores for the Prudent dietary pattern (~48%) approached dietary recommendations for vegetables and fruits consumption of Canada’s Food Guide [43] (approximately 7 servings per day). They also ate more than half of their grain products as whole grains (approximately 4 servings of whole grain products and 2 servings of refined grain products per day). The intakes of meat and alternatives as well as milk and alternatives were around 3 servings per day (data not shown). Thus, high scores for the Prudent dietary pattern were clearly related with Canada’s healthy eating guidelines [43].

It has been observed that high insulin concentrations often relate to insulin resistance [44]. This may indicate a higher risk of insulin resistance among individuals with low Prudent dietary pattern scores which had higher than normal fasting insulin concentrations [44]. Even though individuals with high Western dietary pattern scores had higher fasting glucose than individuals with low scores, these values remained within the normal range (<5.6 mmol/L [45]). Interestingly, individuals with high scores for the Western dietary pattern also had higher systolic and diastolic blood pressure. For individuals with high scores for the Prudent dietary pattern, blood pressure also seemed lower than for individuals with low scores. These associations with dietary patterns and blood pressure have also been observed in other populations [1,2]. For the entire cohort (n = 210), high scores for the Prudent dietary pattern were associated with a more favorable blood lipid profile (data not shown).

Major differences in gene expression profiles were observed between men and women. These differences had been observed previously by our research group [22]. According to the scores for the Prudent dietary pattern, only the B Cell Receptor Signaling pathway was significantly different both for men and women. Sex-specific differences in adipose tissue gene expression have been studied but gene expression differences according to sex in PBMCs are not as well documented [46]. Mechanisms involving sex hormones such as estrogen on transcription factors might partly explain these differences [47]. Moreover, Kawasaki et al. [48] reported fluctuations of the expression of certain genes related to immune and/or inflammatory response according to the menstrual cycle among women. In this study, women were pre-menopausal and the phase of the menstrual cycle was not taken into account, which might explain part of the differences observed.

For participants with high comparatively to low scores for the Prudent dietary pattern, IPA revealed 9 canonical pathways related to immune and/or inflammatory response and 6 to cancer whereas for the Western dietary pattern, 5 pathways related to cancer, 6 to immune and/or inflammatory response and 3 to cardiovascular signaling. Interestingly, predictions made by IPA were pointing towards directional changes in functions which may lead to a decreased risk of cancer among individuals with high scores for the Prudent dietary pattern which is considered a «healthier» pattern (observed among men and women) and changes in functions towards a potential increase of the risk of cancer for individuals with high scores for the Western diet (only observed among men). The PA metabolism also appeared to be modulated with both dietary patterns. PA is mainly formed by the hydrolysis of phosphatidylcholine by phospholipase D [49]. PA is important in heart function and has also been associated with cardiac hypertrophy [50]. For women with high scores for the Prudent dietary pattern, IPA predicted a decrease in tyrosine phosphorylation. Tyrosine phosphorylation may have a protective effect on cancer by reversing the effect of some protein kinase but it may also have a detrimental effect [51]. For example, an increase of phosphorylation in vascular endothelial cadherin tyrosine has been observed after the attachment of invasive breast cancer cells to endothelial cells [52].

Van Dijk et al. [20] have observed a more pro-inflammatory gene expression profile following a diet high in saturated fat compared to a diet high in monounsaturated fat. Saturated fats can modulate the expression of Toll-like receptors (TLRs) therefore increasing the expression of pro-inflammatory genes [53]. In women, according to the Western pattern, the TLRs signaling pathway was different in participants with high versus low scores. Genes within the pathway appeared to be mostly up-regulated which could indicate an increase in the inflammatory response. Conversely, Bowens et al. [54], using shakes containing various amounts of saturated, polyunsaturated and monounsatured fats, observed an increased in the expression of genes involved in TLRs signaling following the high PUFA shake. However, these results were observed in the postprandial state which could explain discrepancies between studies. In addition, the B Cell Receptor Signaling pathway was different according to scores for the Prudent dietary pattern and this was also observed by Bowens et al. with the high PUFA shake [54]. In the present study, the intake of PUFA was higher among individuals with high scores for the Prudent dietary pattern. This pathway is important in humoral immune response and has also been related with chronic lymphocytic leukemia [55]. We hypothesise that the impact of the Prudent dietary pattern on B Cell Receptor Signaling pathway was beneficial due to the predictive results given by IPA and literature on the protective effect of a «healthy» dietary pattern on cancer [56]. When examining pathways common to both dietary patterns, only one was common to the Prudent and Western dietary patterns (among women), the Role of Nuclear Factor of Activated T cells (NFAT) in regulation of the immune response with only one gene overlapping both dietary patterns. The NFAT family of transcription factors induce gene transcription during the immune response. These transcription factors have been linked with cardiac hypertrophy which increases the risk of cardiovascular diseases and have a dual role in cancer acting as a tumor suppressors as well as oncogenes [57,58].

Results observed in this exploratory study support the scientific evidence regarding the beneficial effects of the consumption of a healthy diet and the deleterious impacts of a Western dietary pattern. These results also seem to indicate that gene expression profiles and expression of genes in pathways related to chronic disease are influenced by the presence of a few or more dietary characteristics according to a dietary pattern (high versus low scores). However, due to the small number of participants, these results should be interpreted with caution. In addition, many other factors associated with a healthy or unhealthy lifestyle may impact gene expression. For example, physical activity has an effect on gene expression profiles and was not taken into account in the analyses [59].

## Conclusion

Data retrieved from this nutrigenomic study provide valuable information on biologically relevant pathways that might relate to chronic disease prevention or initiation. Transcriptomics analysis gives us further insights to understand the global effect of dietary patterns on health. In this study, both the Prudent and Western dietary patterns were related to biological pathways associated with cancer, immune and/or inflammatory response and cardiovascular signaling. It appears from these results that the Prudent dietary pattern has a protective effect on cancer initiation or development and the opposite is observed for the Western dietary pattern. However, these data reflect gene expression profile and statistical predictions and need to be confirmed by further research.

## Abbreviations

ApoB: Apolipoprotein B-100; BMI: Body mass index; cRNA: Complementary RNA; CRP: C-reactive protein; DBP: Diastolic blood pressure; FDR: False discovery rate; FFQ: Food frequency questionnaire; GLM: General linear model; GNB5: Guanine nucleotide binding protein (G protein), beta 5; HDL-C: HDL-cholesterol; IPA: Ingenuity pathway analysis; LDL-C: LDL-cholesterol; n-3 PUFA: Omega-3 PUFA; NFAT: Role of nuclear factor of activated T cells; PA: Phosphatidic acid; PBMCs: Peripheral blood mononuclear cells; PCR: Polymerase chain reaction; PPARs: Peroxisome proliferator-activated receptors; RD: Registered dietitian; RIN: RNA integrity number; SAM: Significance analysis of microarrays; SAS: Statistical analysis software; SBP: Systolic blood pressure; TC: Total cholesterol; TLRs: Toll-like receptors.

## Competing interests

The authors declare no competing of interests.

## Authors’ contributions

SL, PC, IR, AMP and MCV designed research; AMP and ABM conducted research; SL, PC, IR, AMP and MCV provided essential reagents or provided essential materials; ABM analyzed data and performed statistical analysis; ABM wrote paper; ABM, SL, PC, IR, AMP and MCV had primary responsibility for final content; All authors read and approved the final manuscript.
